# Characterization and Quantification of Polyphenols in Amazon Grape (*Pourouma cecropiifolia* Martius)

**DOI:** 10.3390/molecules15128543

**Published:** 2010-11-26

**Authors:** Daise Lopes-Lutz, Judith Dettmann, Chamila Nimalaratne, Andreas Schieber

**Affiliations:** Department of Agricultural, Food and Nutritional Science, University of Alberta, 410 Agriculture/Forestry Centre, Edmonton, AB T6G 2P5, Canada

**Keywords:** *Pourouma cecropiifolia* Martius, Amazon grape, anthocyanins, polyphenols, LC-MS

## Abstract

The phenolic profile of Amazon grape fruit (*Pourouma cecropiifolia* Martius) was investigated by high-performance liquid chromatography-electrospray ionization mass spectrometry (HPLC-ESI-MS/MS). For this purpose, suitable extraction and liquid chromatographic methods were developed. Anthocyanins, flavonols and chlorogenic acids were found mainly in the peel. Besides the main anthocyanins, *i.e.* delphinidin 3-glucoside, cyanidin 3-glucoside and cyanidin 3-(6”-malonyl)glucoside, several minor anthocyanins were identified in the peel. Among these, cyanidin 3,5-diglucoside, delphinidin 3-galactoside, cyanidin 3-rutinoside, cyanidin 3-(3”-malonyl)glucoside, malvidin 3-glucoside, pelargonidin 3-glucoside, peonidin 3-glucoside and petunidin 3-glucoside were characterized on the basis of their fragmentation patterns in MS/MS experiments. The total anthocyanin content in the peel was 420.26 ± 3.07 mg kg^-1^ fresh weight. The pulp contained mainly 5-*O*-caffeoylquinic acid (210.39 ± 3.43 mg kg^-1^ fresh weight). Rutin was the predominant flavonol found in Amazon grape (peel 155.45 ± 2.06 mg kg^-1^ fresh weight and pulp 2.64 ± 1.21 mg kg^-1^ fresh weight). Total polyphenols content was higher in the peel than in the pulp.

## 1. Introduction

Fruits are excellent sources of bioactive components, mainly secondary plant metabolites such as carotenoids and polyphenols. Recently, anthocyanins, along with other phenolics, have attracted much interest since they are major antioxidants in our diet and may impart health benefits linked to their antimicrobial, anti-inflammatory, and anticarcinogenic activities, insulin secretion ability, and neuroprotective effects [1]. Interest in the role of antioxidants in human health has prompted research in the field of food science to assess new sources of fruit antioxidants. 

Amazon grape (*Pourouma cecropiifolia* Martius), which belongs to the Moraceae family, is a tropical fruit that has a sweet and juicy flesh with a characteristic flavor which resembles Muscat grape with a mild wintergreen mint aroma. It is the only species of *Pourouma* that is cultivated for the consumption of the fruits in several countries located in the western Amazon Basin since pre-Hispanic times and is also referred to as mapati. The dark purple spherical drupes (2-4 cm) are eaten fresh or processed into jams, jellies, marmalades and wine. It is a dioecious tree that grows to 12 m and starts producing fruits in the third year [2,3]. Previous reports on the fruit composition revealed that the pulp had low acidity and high contents of sugars and minerals such as potassium, calcium and phosphorous. The flavor composition was also investigated and the principal volatile constituents were methyl salicylate and the oxygenated monoterpenes: *cis*-linalool oxide (furan isomer), *trans*-linalool oxide (furan isomer), linalool, *p*-menth-1-en-9-al, α-terpineol and geraniol. These constituents enabled a correlation of the mapati flavor with that of white varieties of *Vitis vinifera*, since the characteristic volatiles of the latter are linalool, furanoid linalool oxides, α-terpineol, β-ionone, geraniol, nerol and citronelol [4]. Very recent *in vitro* studies suggest promising cytotoxic effects of Amazon grape extracts on several cancer cell lines, which however need to be confirmed using *in vivo* experiments [5]. To obtain a more comprehensive knowledge of the composition of this tropical fruit, the aim of the present investigation was to identify and quantify individual phenolic compounds in the peel and pulp of Amazon grape by HPLC-DAD-ESI-MS/MS. 

## 2. Results and Discussion

Because of the presumed benefits to human health and the trend to natural food additives including pigments, there is a constant search for novel sources of anthocyanins. In many cases, studies are not limited to anthocyanins but comprise also other flavonoids and phenolic acids. Using LC-ESI-MS/MS methods, the profile of phenolic compounds in Amazon grape was characterized. The major compounds of each class of polyphenols present were quantified by external calibration using LC-DAD methods. 

The results of Folin-Ciocalteu assay showed that the peel had the highest polyphenols content. Total polyphenols contents were 84.66 ± 1.22 mg gallic acid equivalent in 100 g fresh peel and 8.85 ± 3.74 mg gallic acid equivalent in 100 g fresh pulp. Although this assay is usually employed to determine “total polyphenols” it should be noted that it actually measures the reducing capacity of a sample and, therefore, can be considered another assay for the determination of the antioxidant activity. As a result, the Folin-Ciocalteu assay tends to overestimate the “true” total phenols content due to interference of reducing substances such as ascorbic acid.

**Figure 1:**
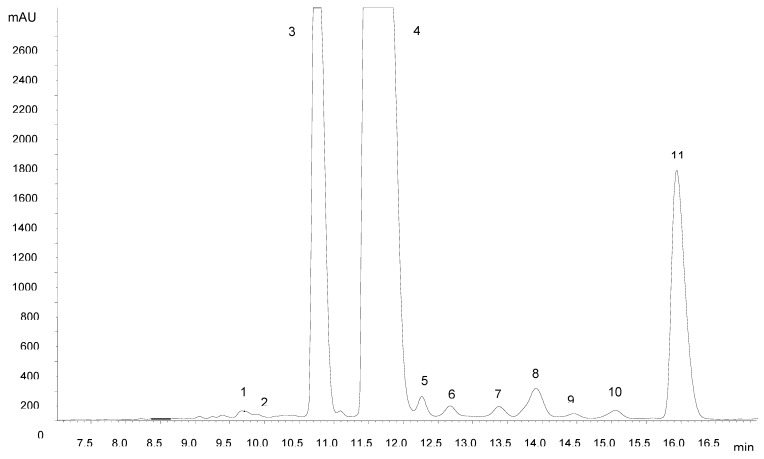
Separation of anthocyanins extracted from Amazon grape peels by HPLC with diode array detection (520 nm).

The amount of anthocyanin pigments measured in the peel was 420.26 ± 3.07 mg kg^-1^ fresh weight. The identification of individual anthocyanins was accomplished by HPLC-DAD-MS/MS analysis. From Figure 1 it can be seen that a satisfactory separation of three major anthocyanins and several minor compounds could be achieved within 20 min. The wavelength extracted chromatogram at 520 nm showed no interfering compounds, allowing the analysis of Amazon grape anthocyanins directly using the extract without partitioning with ethyl acetate. 

The identification of major anthocyanins (Table 1) was based on comparison of their retention times with those of reference compounds and their elution order on reversed-phase C18 columns [6,7]. Peak assignment was confirmed by mass spectrometry. MS^2^ experiments yielded product ions at *m/z* 287 (cyanidin), 303 (delphinidin), 317 (petunidin), 271 (pelargonidin), 301 (peonidin) and 331 (malvidin), in most cases formed after the loss of 162 a.m.u. attributed to a hexose. CID of the [M]^+^ ion at *m/z* 611 resulted in fragments at 449 and 287, corresponding to the aglycone cyanidin and the loss of two hexose moieties. Compounds 8 and 11 had [M]^+^ ions at 535 Da. CID of the molecular ion of compound 11 led to the formation of a predominant fragment at *m/z* 287 and another fragment of lower intensity at *m/z* 449 formed by the loss of 86 a.m.u., which can be ascribed to release of the malonyl moiety. This fragmentation behavior is in accordance with literature data for cyanidin 3-(6”-malonyl)glucoside; the more stable cyanidin 3-(3”-malonyl)glucoside (compound 8) yielded only a product ion at *m/z* 287, showing that the acyl linkage to the 6”-position of the sugar is more labile than the corresponding linkage to the 3”-position [8]. Furthermore, the order of elution of compounds was found to be in accordance with that described previously under reversed-phase conditions [6,7]. Delphinidin 3-glucoside, cyanidin 3-glucoside and cyanidin 3-(6”-malonyl)glucoside have recently been identified in Amazon grape [5].

**Figure 2:**
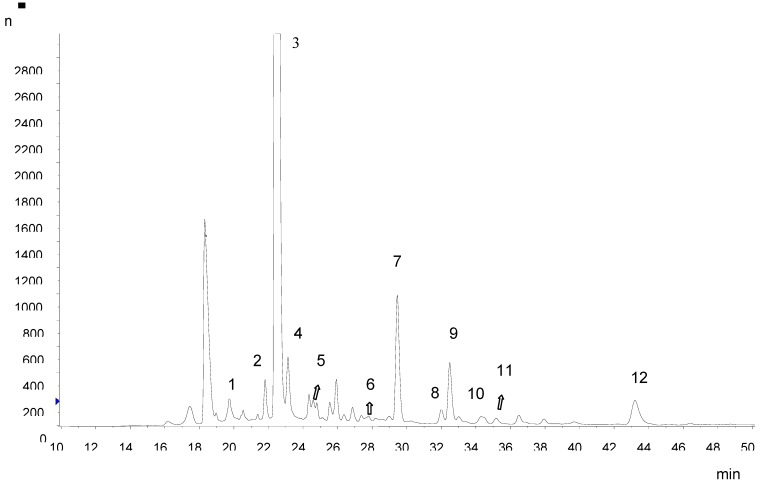
Separation of phenolic acids and other flavonoids extracted from Amazon grape peels by HPLC with diode array detection (320 nm).

Ethyl acetate partition is particularly recommended if other phenolic compounds need to be subsequently analyzed by mass spectrometry. This is due to the large amount of anthocyanins in the peel extract that could make MS assignments difficult. Also, it was necessary to develop a separate HPLC method for the analysis of hydroxycinnamic acid derivatives and flavonols. Hydroxycinnamic acid derivatives were the most abundant group of polyphenols in Amazon grape peel and pulp. Chlorogenic acid (5-*O-*caffeoylquinic acid) was the predominant hydroxycinnamate, with contents of 685.44 ± 5.31 and 210.39 ± 3.43 mg kg^-1^ fresh weight in the peel and pulp, respectively. The LC-MS/MS data showed the presence of neochlorogenic (3-*O-*caffeoylquinic acid), chlorogenic, 4,5-*O-*dicaffeoyl quinic and 5-*O-*feruloyl quinic acids in Amazon grape extracts (Table 2; Figure 2). Chlorogenic acid was identified by comparison of the retention time and MS data with a commercial standard. Neochlorogenic acid, 5-*O-*feruloyl quinic acid and 4,5-*O-*dicaffeoylquinic acid were identified by their elution order under reversed-phase conditions and their MS fragmentation pattern. 3-*O-*Caffeoyl quinic acid gave the same base peak as 5-*O-*caffeoylquinic acid but could be distinguished by a more intense fragment at *m/z* 179 derived from caffeic acid. For 5-*O-*feruloyl quinic acid, the MS^2^ base peak *m/z* 191 was derived from the cinnamic acid moiety. The identification of 4,5-*O-*dicaffeoylquinic acid was based on the similarity of MS^2^ and MS^3^ spectra with those reported by Clifford *et al.* [9]. The ion *m/z* 173 is diagnostic for substitution at position 4. 

Flavonols, flavan-3-ols and a proanthocyanidin dimer were also identified in Amazon grape extracts. Quercetin derivatives were the characteristic flavonols found, rutin being the most abundant. The rutin content in the peel was 155.45 ± 2.06 mg kg^-1^ fresh weight and in the pulp 2.64 ± 1.21 mg kg^-1^ fresh weight. The identification of quercetin derivatives in Amazon grape was based on comparison of their retention times and mass spectrometric data with those of pure standards of rutin, quercetin-3-galactoside and quercetin-3-glucoside, and mango peel extract which was characterized by Schieber *et al.* [10]. Catechin and epicatechin were the only free flavan-3-ols found, and their retention times and mass spectra were compared with those of reference compounds. A proanthocyanidin dimer was tentatively identified based on MS fragments [11].

## 3. Experimental Section

### 3.1. Chemicals

Sodium hydroxide, water (HPLC grade), methanol (HPLC grade), acetonitrile (HLPC grade), formic acid and acetic acid were purchased from Fisher Scientific (Ottawa, ON, Canada). Folin-Ciocalteu reagent (2N), sodium carbonate, gallic acid and polyamide for column chromatography were supplied by Sigma–Aldrich (Oakville, ON, Canada); cyanidin 3-glucoside chloride, cyanidin 3-rutinoside chloride and cyanidin 3,5-diglucoside chloride were obtained from Extrasynthèse (Genay Cedex, France). Chlorogenic acid, (+)-catechin, (-)-epicatechin, rutin, quercetin-3-glucoside, and quercetin-3-galactoside were purchased from ChromaDex (Santa Ana, CA, USA). A C_18_ Sep-Pak cartridge (1 g) was obtained from Waters (Milford, MA, USA). 

### 3.2. Plant material

Ripe fruits (3 kg) were harvested in December 2009 from trees cultivated in Manaus, Amazon, Brazil. The most uniform fruits were separated and frozen at -70 °C until analysis. The average weight of the fruits was 10.4 g (52.3% pulp, 20.1% seed, and 7.4% peel).

### 3.3. Sample preparation

Frozen fruits were peeled manually using a stainless steel knife. Polyphenols were extracted by homogenizing frozen peel (12 g) and frozen pulp (22 g) in 80% aqueous methanol containing 0.1% formic acid (70 mL) for 30 s. The mixture was sonicated for 20 min and centrifuged for 30 min (13,000 *g*, 4 °C). The samples were extracted another three times using the same procedure. For the spectrophotometric determination of total polyphenols, the supernatants were combined, adjusted to a defined volume, and filtered through 0.45 µm filter. For high-performance liquid chromatography analysis, the combined supernatants were evaporated to dryness at 40 °C under vacuum. The residue was dissolved in water containing 0.1% formic acid (pH adjusted to 1.5 with formic acid) and transferred to a polyamide column. The resin (10 g) was eluted with aqueous 0.1% formic acid solution (60 mL) to remove sugars and then with 0.1% formic acid in methanol (200 mL) to recover the polyphenols. The eluate was evaporated at 40 °C under vacuum to remove the organic solvent and the residue dissolved in water containing 0.1% formic acid. A 10 µL aliquot was injected for HPLC analysis of anthocyanins [7].

For the analysis of non-anthocyanin polyphenols, the extraction was performed as described above and the residue obtained after evaporation of methanol was dissolved in water containing 0.1% formic acid (pH adjusted to 1.5 with formic acid). The aqueous solution was extracted four times with 50 mL of ethyl acetate and the organic phase was evaporated to dryness. The residue was dissolved in water and the pH adjusted to 7 with 1 N NaOH solution. It was applied to a C_18_ Sep-Pak cartridge (1 g). After washing with water containing 0.1% formic acid (10 mL), the polyphenol fraction **1** was collected. Polyphenol fraction **2** was recovered after eluting the C_18_ Sep-Pak cartridge with ethyl acetate. Both fractions were evaporated to dryness using a rotary evaporator. The residue was dissolved in 80% aqueous methanol (0.1% formic acid) [8].

### 3.4. Determination of total phenols by the Folin-Ciocalteu assay

The total phenols content of the extracts was measured using a modified colorimetric Folin-Ciocalteu assay as described by Singleton *et al.* [12]. One mL of diluted fruit extract and 1 mL of Folin-Ciocalteu reagent were transferred to a 100 mL volumetric flask. After 3 min, a 20% aqueous solution of sodium carbonate (Na_2_CO_3_, 10 mL) was added, and the flask was made up to volume with distilled water. The absorbance at 765 nm was measured after 1 h, and the measurement was compared to a standard curve of gallic acid. Concentrations were expressed as milligrams of gallic acid equivalents per 100 g of fresh weight ± SD for triplicate fruit extracts.

### 3.5. HPLC analysis of anthocyanins

Anthocyanin analysis was conducted using a 1200 Series Agilent Technologies HPLC (Agilent, Palo Alto, CA, USA) which was equipped with a model G1315D diode array detector (DAD), a model G1379B degasser, a model G1312A binary gradient pump, a model G1329A thermoautosampler and a model G1316A column oven. The reversed-phase separation was performed on a 250 mm × 4.6 mm i.d. Symmetry C18 column, particle size 5 µm (Waters, MA, USA) with a Nova-Pak 4 µm C18 guard column 3.9 × 20 mm operating at 35 °C and at a flow rate of 1.0 mL/min. The compounds were separated with gradient elution of (A) 4.5% aqueous formic acid and (B) 80% acetonitrile in solution A. The gradient program was: 0-15% B (9 min), 15-45% B (22 min), 45-100% B (6 min), 100-0% B (1 min), 2 min 0% B. The injection volume was 10 uL. Monitoring was performed at 520 nm and the diode array detector was set at an acquisition range from 200 nm to 700 nm at a spectral acquisition rate of 1.25 scans s^-1^ (peak width 0.2 min). Quantification was conducted by LC-DAD with external calibration using a set of seven standard dilutions of cyanidin-3-glucoside at 520 nm. A stock solution of 1 mg/mL in 80% methanol in aqueous 0.1% formic acid was prepared and stored at -20 °C. The calibration curves were linear over the range of 1 to 200 µg/mL with a correlation coefficient of ≥ 0.99. For quantification, peak areas were correlated with concentrations in accordance with the calibration curves. Data are reported as means ± standard deviations of triplicate independent analyses.

### 3.6. HPLC analysis of phenolic acids and other flavonoids

The analysis of phenolic acids and flavonols was conducted with a 1200 Series Agilent Technologies HPLC (Agilent, Palo Alto, CA, USA) equipped with a model G1315D diode array detector (DAD), a model G1379B degasser, a model G1312A binary gradient pump, a model G1329A thermoautosampler and a model G1316A column oven. The reversed-phase separation was performed on a 250 mm × 4.6 mm i.d. Luna C18(2) column, particle size 5 µm (Phenomenex, Torrance, CA, USA) with an AQ 4 × 20 mm C_18_ precolumn (Phenomenex) operating at 20 °C and at a flow rate of 0.5 mL/min. The compounds were separated with gradient elution of (A) 2% aqueous acetic acid solution and (B) 0.5% aqueous acetic acid solution and acetonitrile (50:50, v/v). Samples were eluted with the following gradient: 0% B (5 min), 0-40% B (10 min), 40-60% B (40 min), 60-80% B (10 min), 80-100% B (10 min), 100% B (30 min), and 100-0% B (2 min). The injection volume was 20 uL. Monitoring was performed at 280 and 320 nm and the diode array detector was set at an acquisition range from 200 nm to 700 nm at a spectral acquisition rate of 1.25 scans s^-1^ (peak width 0.2 min). Quantification was performed by LC-DAD using external calibration with a set of seven standard dilutions of rutin at 320 nm and chlorogenic acid at 280 nm. Data acquisition, peak integration, and calibrations were performed with Agilent Chemstation software. A stock solution of 1 mg/mL in 100% methanol was prepared and stored at -20 °C. The calibration curves were linear over the range of 1 to 500 µg/mL with a correlation coefficient of ≥ 0.99. For quantification, peak areas were correlated with concentrations in accordance with the calibration curves. Data are reported as means ± standard deviations of triplicate independent analyses.

### 3.7. HPLC-ESI-MS/MS determination of individual polyphenols

The chromatographic system consisted of an Agilent 1200 Series HPLC unit comprising a degasser, binary pump, autosampler, thermostated column compartment, and diode array detector (Agilent Technologies, Palo Alto, CA, USA) connected to a linear ion trap mass spectrometer 4000 QTRAP system (AB Sciex, ON, Canada) which was equipped with an ESI Turbo V™ source. The reversed-phase separation of anthocyanins was performed on a 250 mm × 4.6 mm i.d. Symmetry C18 column, particle size 5 µm (Waters, MA, USA) with a Nova-Pak C18 guard column 4 µm 3.9 × 20 mm using the same conditions as described above. 

For the HPLC-MS analysis of anthocyanins, the effluent from the column after the DAD detector was directly introduced into the electrospray ion source (ESI). The tuning of MS instrument parameters was performed by compound optimization using a 100 ng/mL solution of cyanidin 3-glucoside dissolved in 80% methanol/20% formic acid (0.1%) solution. The optimization of ion source parameters was conducted using a flow rate of 1.0 mL/min. High-purity nitrogen gas (99.995 %) was used as the nebulizing (GS1) and heating gas (GS2). The values for optimum spray voltage, source temperature, GS1, GS2 and curtain gases were +3.0 kV, 600 °C, 40, 40 and 20 psi, respectively.

An information-dependent acquisition (IDA) method, EMS → 4 EPI, was used to profile the anthocyanins in positive mode. The spectra were obtained over a range from *m/z* 50 to 1300 in 1 s. LIT fill time was set at 20 ms. The IDA threshold was set at 100 cps, above which enhanced product ion (EPI) spectra were collected from the eight most intense peaks. The EPI scan rate was 4000 amu s^-1^. Collision-induced dissociation (CID) spectra were acquired using nitrogen as the collision gas under a collision energy of 20 eV (CES15). The other MS parameters used were as follows: declustering potential (DP), 96 V; entrance potential (EP), 10V; and collision exit potential (CXP) 3V. 

The separation of the non-anthocyanin phenolic compounds was performed on a 250 mm × 4.6 mm i.d. Luna C18(2) column, particle size 5 µm (Phenomenex, Torrance, CA, USA), using the same conditions described above. The mass spectra were recorded in negative mode; the flow rate was maintained at 0.5 mL/min with the pneumatically assisted electrospray probe using high-purity nitrogen gas (99.995%) as the nebulizing (GS1) and heating gas (GS2). The values for optimum spray voltage, source temperature, GS1, GS2, and curtain gases were -4 kV, 600 °C, and 50, 30, and 25 psi, respectively. An information-dependent acquisition (IDA) method, EMS → 4 EPI, was used to identify phenolic compounds. Both Q1 and Q3 were operated at low and unit mass resolution. The spectra were obtained over a range from *m/z* 50 to 1300 in 1 s. LIT fill time was set at 20 ms. The IDA threshold was set at 100 cps, above which enhanced product ion (EPI) spectra were collected from the eight most intense peaks. The EPI scan rate was 4000 amu s^-1^. Collision-induced dissociation (CID) spectra were acquired using nitrogen as collision gas under a collision energy of -20 eV. The other MS parameters were as follows: declustering potential (DP), -70 V; entrance potential (EP), -10V; and collision exit potential (CXP), -7V. Data acquisition was interfaced to a computer workstation Analyst 1.5 (Applied Biosystems, CA, USA).

## 4. Conclusions

This study clearly shows that LC-ESI-MS/MS is a powerful technique enabling fast separation and characterization of polyphenols reported herein for the first time in Amazon grape. The high sensitivity of this hyphenated technique allowed expanding previous characterization of Amazon grape to additional eight minor anthocyanins, four quercetin glycosides, catechin, epicatechin, procyanidin B and four hydroxycinnamic acid derivatives. In this study, only peel and pulp samples were investigated for their profile of phenolic compounds. It would be desirable to characterize also the polyphenols present in the seeds, which constitute approximately 20% of the total fruit weight and are by-products of processing, to assess their potential as a source of natural bioactive components.

## Figures and Tables

**Table 1:** Identification and contents of anthocyanins in Amazon grape peel.

Peak no.	*t*_R_ (min)	[M]^+^ (*m/z*)	MS/MS (*m/z*)	Identity	Content ^a^ (mg/kg fresh wt)
1	9.62	611	449/287	cyanidin 3,5-diglucoside	1.53 ± 0.82
2	9.90	465	303	delphinidin 3-galactoside	0.56 ± 0.74
3	10.82	465	303	delphinidin 3-glucoside	104.42 ± 2.45
4	11.75	449	287	cyanidin 3-glucoside	244.57 ± 2.13
5	12.26	595	449/287	cyanidin 3-rutinoside	3.03 ± 0.85
6	12.68	479	317	petunidin 3-glucoside	0.94 ± 0.56
7	13.35	433	271	pelargonidin 3-glucoside	0.69 ± 0.54
8	13.89	535	287	cyanidin 3-(3”-malonyl)glucoside	7.10 ± 0.87
9	14.45	463	301	peonidin 3-glucoside	3.02 ± 0.13
10	15.03	493	331	malvidin 3-glucoside	4.06 ± 0.52
11	16.01	535	449/287	cyanidin 3-(6”-malonyl)glucoside	50.36 ± 2.46

^a^ Content calculated as cyanidin-3-glucoside equivalent.

**Table 2:** Identification of polyphenols in Amazon grape peel and pulp.

Peak no.	*t*_R_ (min)	[M-H]^-^ (*m/z*)	MS/MS (*m/z*)	Identity	Peel	Pulp
1	19.93	353	191/179	3*-O-*caffeoyl quinic acid (neochlorogenic acid)	+	+
2	22.10	289	245/205/179	catechin	+	
3	22.45	353	191	5-*O-*caffeoyl quinic acid (chlorogenic acid)	+	+
4	23.13	577	425	procyanidin B	+	+
5	24.64	289	245/205/179	epicatechin	+	+
6	27.72	367	191	5-*O-*feruloyl quinic acid	+	+
7	29.46	609	301	rutin	+	+
8	31.12	463	301	quercetin 3-galactoside	+	+
9	32.49	463	301	quercetin 3-glucoside	+	+
10	34.4	433	301	quercetin 3-xyloside	+	
11	35.18	433	301	quercetin 3-arabinopyranoside	+	
12	43.21	515	353/179/173/135	4,5-*O-*dicaffeoyl quinic acid	+	+
